# Mesoscopic Analysis of Rounded and Hybrid Aggregates in Recycled Rubber Concrete

**DOI:** 10.3390/ma16196600

**Published:** 2023-10-08

**Authors:** Mahmoud M. A. Kamel, Yu Fu, Xiaowei Feng, Yijiang Peng

**Affiliations:** 1 Key Laboratory of Urban Security and Disaster Engineering, Ministry of Education, Beijing University of Technology, Beijing 100124, China; mahmoudmohamed.kamel@polimi.it; 2 Department of Architecture, Built Environment and Construction Engineering, Politecnico di Milano, 20133 Milano, Italy; 3 China Institute of Building Standard Design Research, Beijing 100013, China; 4 School of Mines, China University of Mining and Technology, Xuzhou 221116, China; fengxiaowei@cumt.edu.cn

**Keywords:** base force element method (BFEM), recycled rubber concrete (RRC), complementary energy principle, random aggregate model, meso-damage analysis

## Abstract

Recycled rubber concrete (RRC), a sustainable building material, provides a solution to the environmental issues posed by rubber waste. This research introduces a sophisticated hybrid random aggregate model for RRC. The model is established by combining convex polygon aggregates and rounded rubber co-casting schemes with supplemental tools developed in MATLAB and Fortran for processing. Numerical analyses, based on the base force element method (BFEM) of the complementary energy principle, are performed on RRC’s uniaxial tensile and compressive behaviors using the proposed aggregate models. This study identified the interfacial transition zone (ITZ) around the rubber as RRC’s weakest area. Here, cracks originate and progress to the aggregate, leading to widespread cracking. Primary cracks form perpendicular to the load under tension, whereas bifurcated cracks result from compression, echoing conventional concrete’s failure mechanisms. Additionally, the hybrid aggregate model outperformed the rounded aggregate model, exhibiting closer peak strengths and more accurate aggregate shapes. The method’s validity is supported by experimental findings, resulting In detailed stress–strain curves and damage contour diagrams.

## 1. Introduction

The significance of recycled rubber concrete (RRC) cannot be overstated. In the recent past, the combined environmental, structural, and economic benefits of recycling waste tires in civil engineering applications have attracted significant attention from scholars and practitioners alike [1,2,3,4]. Recycled rubber concrete, which integrates waste tire rubber particles into traditional concrete, has emerged as a key solution to the escalating problem of waste tire disposal [5,6,7].

Experimental research on RRC has made substantial progress in recent years. Various studies have delved into the mechanical properties, durability, and rheological behavior of RRC, revealing its potential for diverse applications [8,9,10,11]. For instance, the inclusion of rubber has been found to reduce the density, compressive strength, and modulus of elasticity while enhancing the ductility and energy absorption capacity of concrete [12,13,14]. Furthermore, research has indicated that the nature of these alterations is significantly influenced by the rubber content, rubber particle size, and the type of binder used [15,16,17]. However, the understanding of the mesoscopic behavior of RRC, especially that with circular and convex aggregate shapes, remains in its infancy [18,19].

Parallel to experimental endeavors, numerical simulations have also offered valuable insights into the behavior of RRC [20,21,22,23]. This approach provides a granular view of the interactions between aggregates, rubber particles, and the cementitious matrix [24,25,26]. Traditional commercial finite element software, while powerful, is sometimes marred by challenges such as computational inefficiency and mesh sensitivity, especially when modeling complex materials like RRC [18,27,28,29]. The principle of complementary energy, which is a cornerstone for many advanced modeling techniques, has seen its own share of research and development [30,31,32,33]. This principle has led to a better understanding of material behavior, paving the way for more accurate models of real-world behaviors [34].

The base force element method (BFEM) has gained traction within the complementary energy principle domain [35]. Expanding upon Gao Yuchen’s base force concept [36], Peng et al. [35] proposed a mid-node plane element model, leading to refined equations for the element stiffness matrix through the Lagrange multiplier method. They subsequently validated the method’s accuracy for different deformation scales against traditional analytical solutions, showing its broad applicability even in mesh divisions of varied shapes with minimal sensitivity to distortions. Post 2013, Peng et al. employed this method in concrete sectors, revealing insights into its meso-structure, mechanics, and fracture behavior [37]. The findings were further supported by subsequent studies, especially those exploring prismatic recycled concrete’s damage mechanisms and the role of aggregates. To sum up, the BFEM offers an effective solution for computational efficiency and mesh sensitivity, especially in heterogeneous materials like RRC. Additionally, it is important for the research community to improve their understanding of the mesoscopic behavior of RRC due to its potential applications and benefits.

This study employs the residual-energy-based principle of the BFEM for an in-depth microscopic examination of RRC. It offers a novel approach to microscopic damage analysis. This research encompasses simulating the genuine shape of RRC’s coarse aggregate using convex polygons for coarse aggregates. Through numerical simulations, this model’s validity and precision are benchmarked against both the random rounded aggregate model and empirical outcomes. In rubber concrete, the weakest point lies in the ITZ (interfacial transition zone) around the rubber. This area tends to develop small cracks that eventually extend into the aggregate, leading to larger cracks. Primary cracks that are perpendicular to the load are caused by tension, while bifurcated cracks similar to those in conventional concrete are caused by compression.

## 2. Two-Dimensional Mesoscopic Model of Recycled Rubber Concrete

### 2.1. Tensor Expression of Element Compliance Matrix

**Figure 1:**
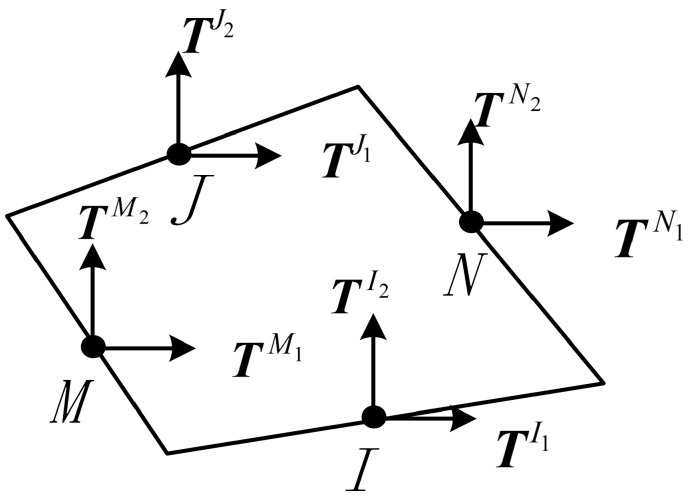
Planar four-node element.

The element of the BFEM is a type of structural element that incorporates mid-side nodes. The governing equations are formulated based on the complementary energy principle, without mandating interpolation functions, which is further elaborated in the literature [35]. The four-node planar fundamental nodal force element is shown in Figure 1, where *I*, *J*, *M*, and *N* are the midpoints of the four sides, and $T^{I},T^{J},T^{M},{\mathrm{and}T}^{N}$ are the resultant forces acting on the midpoints of each side.

The explicit expression of the compliance matrix of the element $C_{IJ}$ can be formulated as follows [35]:

(1) $$C_{IJ}=\frac{1+\upsilon }{EA}\left[r_{IJ}U-\frac{\upsilon }{1+\upsilon }R_{I}⊗R_{J})](I,J=1,2,3,4\right)$$

where E represents the modulus of elasticity; υ represents Poisson’s ratio; *A* is the area of the element; ***U*** is the element tensor; $R_{I}$ and $R_{J}$ are the radius vectors from the origin O to the midpoints *I* and *J***,** respectively; and $r_{IJ}$ represents the dot product of $R_{I}$ and $R_{J}$.

In the equation, *I* and *J*, respectively, represent the *I*-th edge and the *J*-th edge of the element.

For the Cartesian coordinate system, the expression is

(2) $$U=e_{1}⊗e_{1}+e_{2}⊗e_{2}$$

where $e_{1}$ and $e_{2}$ are the direction vectors in directions 1 and 2, respectively.

Therefore, the expanded form of the compliance matrix $C_{IJ}$ is

(3) $$\begin{array}{ll} C_{IJ}= & \frac{1+\upsilon }{EA}[\left(\frac{1}{1+\upsilon }R_{I1}R_{J1}+R_{I2}R_{J2}\right)e_{1}⊗e_{1}-\frac{\upsilon }{1+\upsilon }R_{I1}R_{J2}e_{1}⊗e_{2}\\ & -\frac{\upsilon }{1+\upsilon }R_{I2}R_{J1}e_{2}⊗e_{1}+\left(R_{I1}R_{J1}+\frac{1}{1+\upsilon }R_{I2}R_{J2}\right)e_{2}⊗e_{2}] \end{array}$$

The simplified expression is

(4) $$C_{IJ}=C_{I1J1}e_{1}⊗e_{1}+C_{I1J2}e_{1}⊗e_{2}+C_{I2J1}e_{2}⊗e_{1}+C_{I2J2}e_{2}⊗e_{2}$$

The matrix form of the compliance matrix of the element is

(5) $$C_{IJ}=\frac{1}{EA}\left[\begin{array}{cc} R_{I1}R_{J1}+\left(1+\upsilon \right)R_{I2}R_{J2} & -\upsilon R_{I1}R_{J2}\\ -\upsilon R_{I2}R_{J1} & \left(1+\upsilon \right)R_{I1}R_{J1}+R_{I2}R_{J2} \end{array}](I,J=1,2,3,4\right)$$

Equation (5) is the expression for plane stress problems.

**Figure 2:**
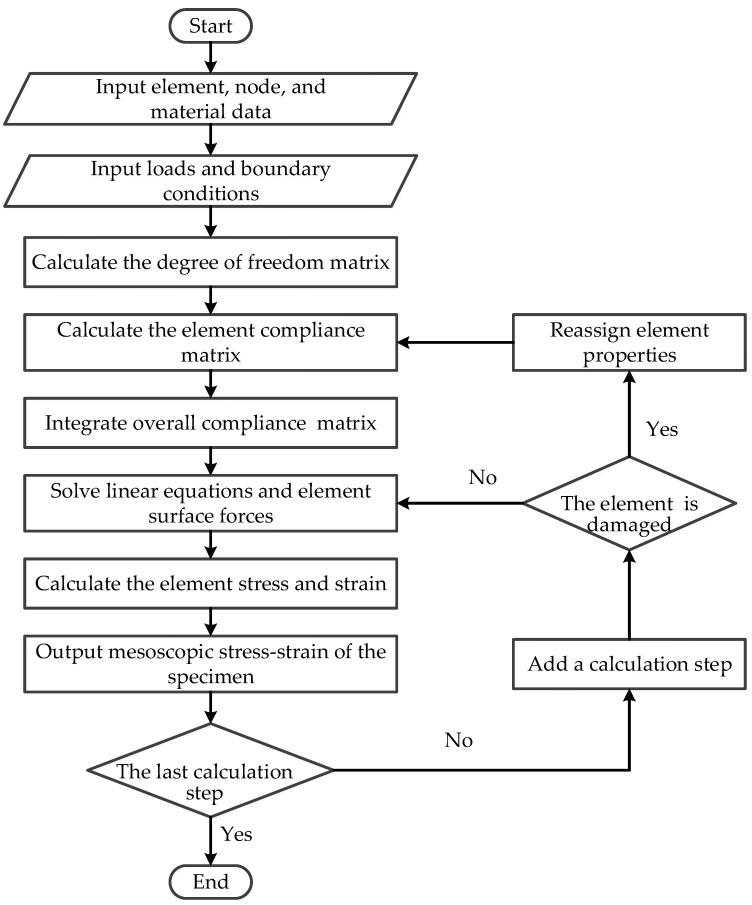
The main program flow chart of the BFEM.

The main program algorithm for the two-dimensional BFEM of the complementary energy principle is shown in Figure 2. The formula of the flow chart is detailed in references [35,37].

### 2.2. Random Rounded Aggregate Model of Recycled Rubber Concrete

**Figure 3:**
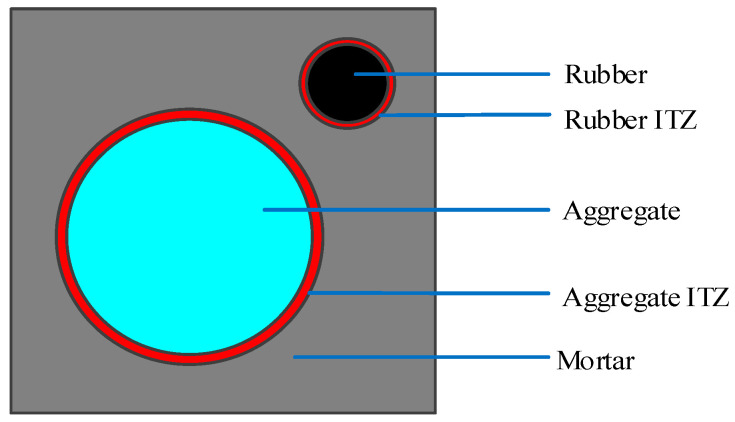
Mesostructure of recycled rubber concrete rounded aggregate.

The random aggregate model is a model that connects the macro and micro levels of recycled rubber concrete (RRC), representing it in the form of different media. The mechanical properties of the macro-material are analyzed by assigning different material properties to each medium. In this research, RRC is represented as a five-phase media, namely coarse aggregate, rubber particles, mortar, the ITZ between mortar and coarse aggregate, and the ITZ between mortar and rubber. In this section, the coarse aggregate and rubber particles are simplified into circles of different sizes, as shown in Figure 3.

Aggregates in concrete are classified into coarse aggregates (particle size greater than 5 mm) and fine aggregates (particle size smaller than 5 mm), with the latter including rubber particles usually considered as fine aggregate. In this study, cement and fine aggregates are treated as cement mortar. And the range of coarse aggregate particle size in the first aggregate grading concrete is 5–20 mm, in which the maximum particle size is 20 mm. The first aggregate grading concrete is adopted in this numerical simulation. In the calculation, the particle size of coarse aggregate is in the range of 5–20 mm, and the representative particle size is taken as 17.5 mm, 12.5 mm, and 7.5 mm. Table 1 presents a detailed analysis of the particle sizes and corresponding particle count within the range observed in the recycled rubber concrete specimen. In the analysis of concrete cross-sections, the areal fraction of coarse aggregate generally ranges from 30% to 60% [38]. This range can be affected by the mix design proportions, the geometric properties of the aggregate particles, and the compaction level of the concrete. In the model presented, the cross-sectional area covered by the coarse aggregate is approximately 32% of the total area.

By implementing the Fuller grading formula, Walraven [39] was able to convert the 3D aggregate grading formula into a 2D equivalent, which facilitated the computation of aggregate particles in two-dimensional RRC. The volume ratio of fine aggregate is derived from experimental data on the apparent density of fine aggregates, with rubber of various particle sizes replacing the same volume of fine aggregates.

The Monte Carlo method [37] is used to compile the FORTRAN program first, and then the pseudorandom number is generated by computer. Then, the position of the coarse aggregate in the center of the circle is determined according to the pseudorandom number and the size of the specimen. The procedure for aggregate distribution is delineated as follows:

- The predetermined dimensions of the rubber concrete specimen are employed to establish the boundaries and the coordinate system.
- Utilizing the Monte Carlo method, uniformly distributed random numbers R and E within the interval (0,1) are procured.
- These two random numbers facilitate the derivation of the coordinates $\left(x_{n},y_{n}\right)$ for aggregate placement, represented by the equation
  (6) $$\left\{ \begin{array}{l} x_{n}=R_{n}\times b\\ y_{n}=E_{n}\times h \end{array}\right.$$
  where b denotes the cross-sectional width of the specimen and h represents its cross-sectional height.
- With the derived coordinates serving as the centroid, the aggregate is strategically positioned. It is imperative to ensure the aggregate remains within the specimen’s dimensional confines. Concurrently, the stipulation demands that the distance between centers of adjacent aggregates surpasses 1.1 times the aggregate diameters’ cumulative value.

**Figure 4:**
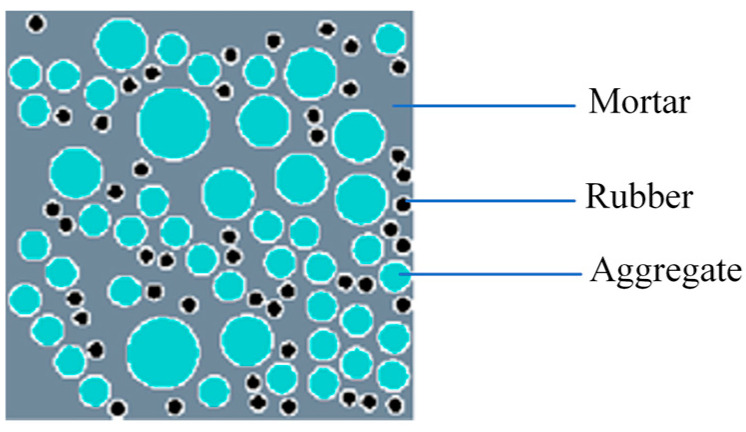
Random round aggregate model of recycled rubber concrete.

The approach of obtaining the random round aggregate model of RRC involves the preparation of an aggregate placing program utilizing the Monte Carlo method, as depicted in Figure 4.

### 2.3. Hybrid Random Aggregate Model of Recycled Rubber Concrete

**Figure 5:**
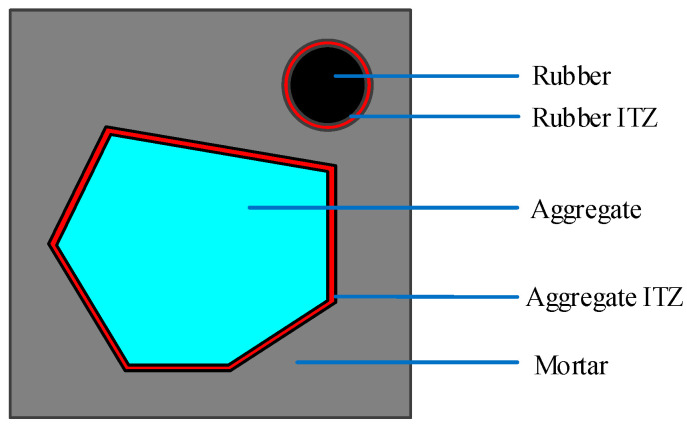
Mesostructure of recycled rubber concrete convex aggregate.

Coarse aggregate’s shape is a vital factor in concrete’s mechanical properties. The existing models of RRC with random aggregate typically simplify the aggregate into a rounded shape [19,25]. Although this simplification is reasonable to some extent, it still deviates significantly from the actual shape of the aggregate. To enhance the model’s authenticity, in this section, the rounded coarse aggregate is modified into a convex polygonal coarse aggregate, based on the method of generating polygonal aggregate [40], while maintaining the rubber particles in a rounded shape. This approach results in a hybrid random aggregate model featuring both convex polygonal coarse aggregate and rounded rubber particles. The mesostructure diagram is shown in Figure 5. Among the various materials, rubber is regarded as fine aggregate.

**Figure 6:**
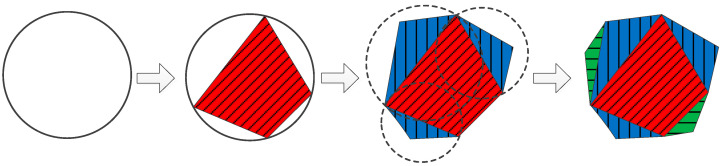
Polygonal aggregate generation process.

In the modeling of coarse aggregate, varying numbers of points are generated on the aggregate’s boundary, forming a polygonal base framework. Additionally, the number of base points for large aggregates exceeds that of small ones, guided by [40]. Once this framework is generated, it expands according to the difference in area between the base frame and the corresponding rounded aggregate, prioritizing longer edges for the outward extension. This expansion must adhere to constraints regarding dimension range, edge length, polygon convexity, and overlap with other aggregates and rubbers. Extension terminates when the polygon’s area matches or exceeds the corresponding rounded area, with the generation process depicted in Figure 6.

**Figure 7:**
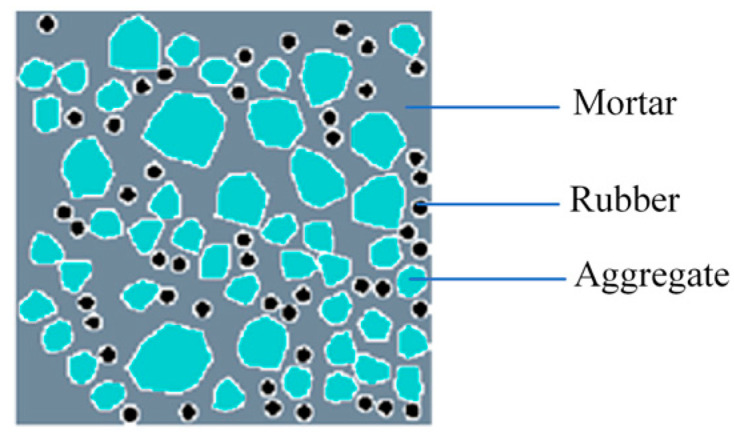
Hybrid random aggregate model of recycled rubber concrete.

This study details a program transforming each coarse aggregate into a convex polygon aggregate, resulting in a hybrid random model (Figure 7), with the aggregate grading being consistent across both models and differing only in shape. This approach, which focuses on the number of vertices in the basic framework and the extension radius, yields more irregular convex aggregates with fewer vertices and larger extension radii.

From the above method of generating polygonal aggregates, the shape of the aggregates is mainly determined by the number of vertices in the basic framework and the extension radius. The fewer the vertices and the larger the extension radius, the more irregular the convex aggregates.

### 2.4. Mesh Generation and Element Attribute Determination

**Figure 8:**
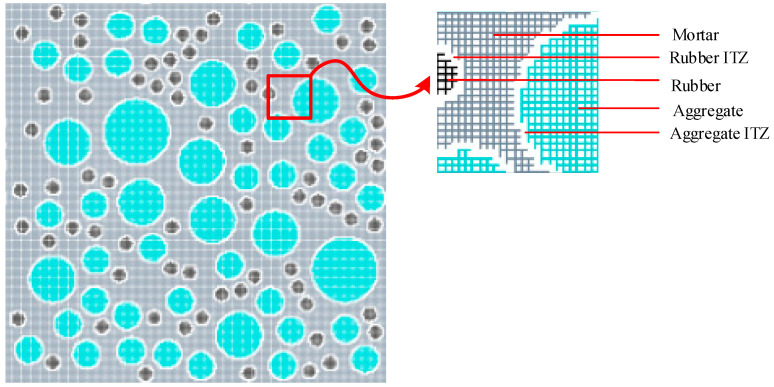
Recycled rubber concrete projection mesh model.

Finite element mesh generation is a critical stage for computation that requires a careful balance between precision and efficiency. In this work, a regular quadrilateral mesh with edge-center nodes and a partition step of 0.5 mm is implemented. This mesh is subsequently projected onto the random aggregate model, with attributes of each mesh determined by the relationship between node positions and aggregates. The meshes are defined as either aggregate, rubber, or mortar, depending on node location within these regions, or as an ITZ mesh when nodes fall between aggregate and mortar or rubber and mortar. The uniformly divided RRC projection mesh model is shown in Figure 8.

## 3. Damage Constitutive Model of Recycled Rubber Concrete Material

The constitutive model of a material reflects the mechanical properties of the material and can determine the stress and strain of the material under external forces. In order to use the finite element method for mesoscale analysis of the established projection mesh model, a suitable constitutive model needs to be found for each phase medium.

This section introduces the bilinear damage constitutive model, multi-linear damage constitutive model, and the rubber constitutive model. Recycled rubber concrete is a composite material consisting of five phases, and the constitutive models used for each phase will be discussed separately in the following.

**Figure 9:**
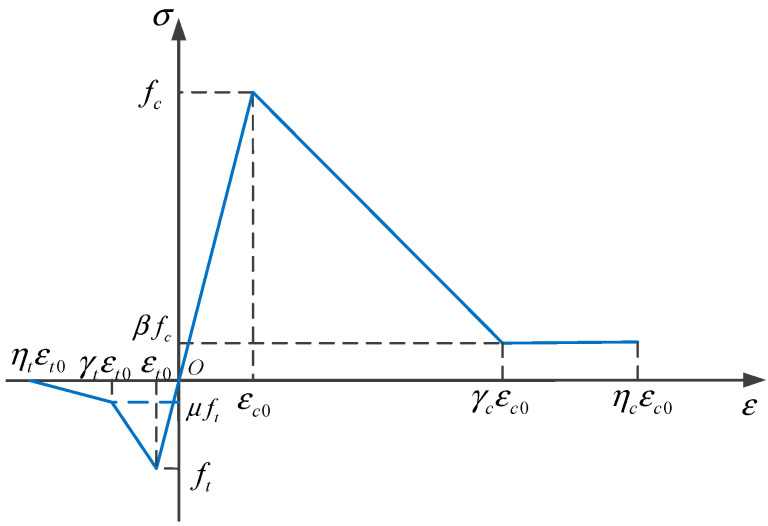
Bilinear damage model.

In RRC, coarse aggregate adopts a bilinear constitutive model [37], as shown in Figure 9.

The bilinear damage model uses the constitutive relationship of isotropic elastic damage mechanics to describe the mechanical properties of concrete materials.

The tensile damage factor *d_t_* and compressive damage *d_c_* [37] are defined as follows:

(7) $$d_{t}=\{\begin{array}{ccc} 0 & & \varepsilon \leq \varepsilon_{t0}\\ 1-\frac{\gamma_{t}-\mu }{\gamma_{t}-1}\frac{\varepsilon_{t0}}{\varepsilon }+\frac{1-\mu }{\gamma_{t}-1} & & \varepsilon_{t0}<\varepsilon \leq \gamma_{t}\varepsilon_{t0}\\ 1-\frac{\mu \cdot \eta_{t}}{\eta_{t}-\gamma_{t}}\frac{\varepsilon_{t0}}{\varepsilon }+\frac{\mu }{\eta_{t}-\gamma_{t}} & & \gamma_{t}\varepsilon_{t0}<\varepsilon \leq \eta_{t}\varepsilon_{t0}\\ 1 & & \varepsilon >\eta_{t}\varepsilon_{t0} \end{array}$$

(8) $$d_{c}=\{\begin{array}{ccc} 0 & & \varepsilon \leq \varepsilon_{c0}\\ 1-\frac{\gamma_{c}-\beta }{\gamma_{c}-1}\frac{\varepsilon_{c0}}{\varepsilon }+\frac{1-\beta }{\gamma_{c}-1} & & \varepsilon_{c0}<\varepsilon \leq \gamma_{c}\varepsilon_{c0}\\ 1-\beta \frac{\varepsilon_{c0}}{\varepsilon } & & \gamma_{c}\varepsilon_{c0}<\varepsilon \leq \eta_{c}\varepsilon_{c0}\\ 1 & & \varepsilon >\eta_{c}\varepsilon_{c0} \end{array}$$

The bilinear damage constitutive simplifies the material stress–strain curve by approximating the ascending and descending segments as straight lines. $f_{c}$ is the compressive strength of the material; $f_{t}$ is the tensile strength of the material; $\varepsilon_{ο}$ is the peak strain; β and μ are the compressive and tensile residual strength coefficients of the material; $\gamma_{c}$ and $\gamma_{t}$ are the residual strain coefficients of the material; and $\eta_{t}$ and $\eta_{t}$ are the limit strain coefficients of the material. The subscript *t* stands for tensile, and the subscript *c* stands for compressive. ε is the maximum principal strain under the current loading state.

The mortar and ITZ in RRC exhibit strong non-linear characteristics near peak stress. In order to better simulate their mechanical properties, a multi-linear damage constitutive model [37] is used to simulate the mortar and ITZ, thus more realistically modeling the damage process of the material.

In the multi-linear damage constitutive model, the tensile damage factor *d_t_* and compressive damage factor *d_c_* of the material are [37]

(9) $$d_{t}=\{\begin{array}{ccc} 0 & & \varepsilon \leq \varepsilon_{t0}\\ 1-\frac{\gamma_{t}-\mu }{\gamma_{t}-1}\frac{\varepsilon_{t0}}{\varepsilon }+\frac{1-\mu }{\gamma_{t}-1} & & \varepsilon_{t0}<\varepsilon \leq \gamma_{t}\varepsilon_{t0}\\ 1-\frac{\mu \cdot \eta_{t}}{\eta_{t}-\gamma_{t}}\frac{\varepsilon_{t0}}{\varepsilon }+\frac{\mu }{\eta_{t}-\gamma_{t}} & & \gamma_{t}\varepsilon_{t0}<\varepsilon \leq \eta_{t}\varepsilon_{t0}\\ 1 & & \varepsilon >\eta_{t}\varepsilon_{t0} \end{array}$$

(10) $$d_{c}=\{\begin{array}{ccc} 1-\frac{\alpha }{\lambda } & & \varepsilon \leq \lambda \varepsilon_{c0}\\ 1-\frac{1-\alpha }{1-\lambda }\frac{\varepsilon -\lambda \varepsilon_{co}}{\varepsilon }-\alpha \frac{\varepsilon_{co}}{\varepsilon } & & \lambda \varepsilon_{c0}<\varepsilon \leq \varepsilon_{c0}\\ 1-\frac{1-\beta }{1-\gamma }\frac{\varepsilon -\varepsilon_{co}}{\varepsilon }-\frac{\varepsilon_{co}}{\varepsilon } & & \varepsilon_{c0}<\varepsilon \leq \gamma \varepsilon_{c0}\\ 1-\frac{\beta \varepsilon_{c0}}{\varepsilon } & & \gamma \varepsilon_{c0}<\varepsilon \leq \eta \varepsilon_{c0}\\ 1 & & \varepsilon >\eta \varepsilon_{c0} \end{array}$$

**Figure 10:**
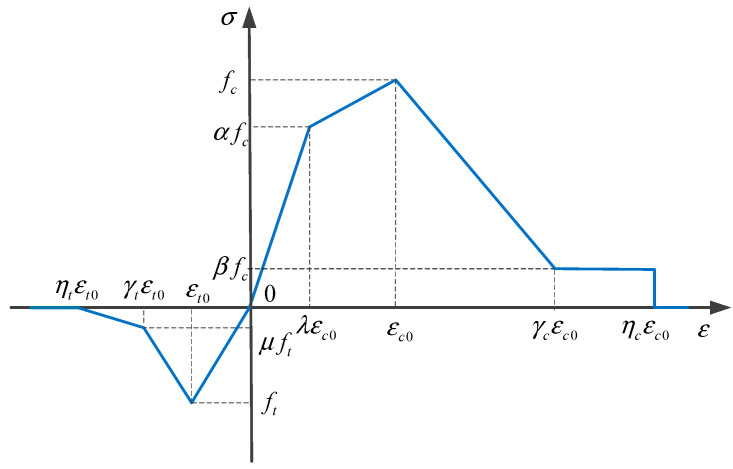
Multi-linear mechanical constitutive model of materials.

In these equations, λ is the elastic strain coefficient, α is the elastic compressive strength coefficient, and the other coefficients remain unchanged compared to the bilinear model. The multi-linear damage constitutive model [37] is shown in Figure 10.

Rubber’s incorporation into concrete significantly influences the mechanical properties due to its reduced strength and weaker bond with mortar compared to coarse aggregate. Consequently, choosing the appropriate rubber constitutive model is vital. Mainstream models are categorized into hyperelastic and linear elastic models. Rugsaj and Suvanjumrat [41] found the linear elastic model to be more accurate for simulating tires, particularly under small deformations. Since the rubber particles implemented into the concrete in the experiments were mainly crushed by tires and the deformation of the rubber in the concrete is small, the rubber is considered as an elastic material. Therefore, this study uses a linear elastic constitutive model to simulate rubber, which only provides the elastic modulus and does not consider the damage of rubber. The tensile strength and compressive strength are not provided in Table 2.

## 4. Numerical Simulation of Recycled Rubber Concrete

### 4.1. Model Parameter Selection

In this study, the 100 mm × 100 mm × 100 mm cube specimen is simplified into a 100 mm × 100 mm two-dimensional plane model, and the RRC random rounded aggregate model and the hybrid random aggregate model are separately established. The ITZ is a key component of concrete mesoscopic-scale research. Studies have shown that the ITZ thickness in concrete is only 10 μm–50 μm. In numerical simulation, when the thickness of the ITZ is taken as 0.1–0.8 mm, the impact on mechanical properties is minimal [37]. Therefore, in this section, the model is divided into 0.5 mm × 0.5 mm quadrilateral meshes. The aggregate adopts a bilinear damage constitutive model, while the mortar and ITZ adopt a multi-line damage constitutive model, and the rubber adopts a linear elastic constitutive model. The material parameters of the aggregate and rubber particles are determined according to the literature [37,42,43], and the material parameters of the mortar are determined according to the empirical formula [44]. First, the water–cement ratio of RRC in the experiment [45] is substituted into the formula to obtain the compressive strength, and then the compressive strength is substituted into formulas to obtain the modulus of elasticity and tensile strength of the mortar. According to reference [46], the parameters of the aggregate and mortar ITZ are 65% of the mortar material parameters, and the parameters of the rubber and mortar interface are 35% of the mortar material parameters. The performance Poisson’s ratio of the interfacial transition zone (ITZ) is difficult to test on an experimental scale, so the determination of the simulation parameter Poisson’s ratio for the ITZ is difficult to determine. Usually, the performance of the ITZ is approximated by the weak mortar composition, and researchers use the percentage of mortar to study and judge the performance of the ITZ. In the finite element calculation, the Poisson ratio of the ITZ is usually approximately 0.2 [43]. The parameter selection of the constitutive model is obtained using inverse inference according to the strength and elastic modulus of each phase medium. The material parameters and constitutive parameters are shown in Table 2 and Table 3.

(11) $$f_{c}=\left(\frac{1}{w/c}-0.5\right)/0.047$$

(12) $$E_{m}=1000\left(7.7\ln\left(f_{c}\right)-5.5\right)$$

(13) $$f_{t}=1.4\ln\left(f_{c}\right)-1.5$$

In above equations [46], $E_{m}$ is the modulus of elasticity of the mortar; $f_{t}$ is the tensile strength of the mortar; $f_{c}$ is the compressive strength of the mortar; and wc is the water–cement ratio.

The two-dimensional random aggregate model with dimensions of 100 mm×100 mm has a maximum coarse aggregate particle size of 20 mm and a minimum particle size of 5 mm. The coarse aggregate is divided into three particle size ranges. The number of aggregates in each particle size range is shown in Table 1.

Based on the mix proportion (${MP}_{FA}$) of fine aggregates in the concrete and the apparent density ($\rho_{FA}$) of fine aggregate in the experimental data [45], the volume ratio of fine aggregates in the concrete mix ($P_{k}$) can be calculated as

(14) $$P_{k}=\frac{{MP}_{FA}(Kg/m^{3})}{\rho_{FA}(Kg/m^{3})}=\frac{780}{2650}=0.3$$

The rubber particle size is used to replace 20% of the volume of fine aggregates. The number of rubber particles ($n_{x}$) is calculated using Formula (15).

(15) $$n_{x}=P_{k}\times c\times A/A_{i}$$

where *c* is the mixing ratio of rubber, A is the cross-sectional area of the rubber concrete, and $A_{i}$ is the area of the rubber particle of that size.

The RRC specimen measuring 100 mm × 100 mm contains 47 rubber particles characterized by a diameter of 4 mm.

According to the Monte Carlo method, coarse aggregates and rubber particles are randomly placed in the model, the mesh is divided, and parameters are assigned to the mesh elements.

### 4.2. Uniaxial Tension and Compression Loading Models

Two types of mesoscopic random aggregate models are established to verify the accuracy of the numerical model based on the base force element method (BFEM) of the complementary energy principle. In addition, the static tensile and compressive mechanical properties and damage failure process of RRC are studied. Uniaxial tensile and compression numerical simulations are performed on a 100 mm × 100 mm × 100 mm RRC cubic specimen with a rubber particle size of 4 mm and a rubber content of 20%.

When the proportions of aggregates and rubber particles are kept constant, the RRC is represented using two modeling approaches: random rounded aggregate models and hybrid random aggregate models. Three distinct random distributions are conducted to mitigate the effects of aggregate randomness on the mechanical attributes, with unique random numbers input for each group.

**Figure 11:**
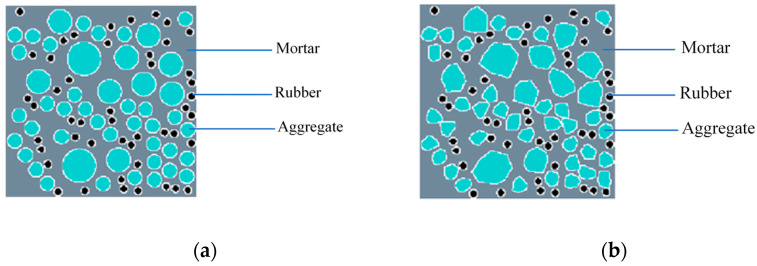
Model diagram: (**a**) rounded aggregate model; (**b**) hybrid aggregate model.

Figure 11 illustrates schematic diagrams of the models, containing both types of random aggregate models. In the figure, gray represents the mortar, cyan represents the aggregates, black represents the rubber particles, and white represents the ITZ.

**Figure 12:**
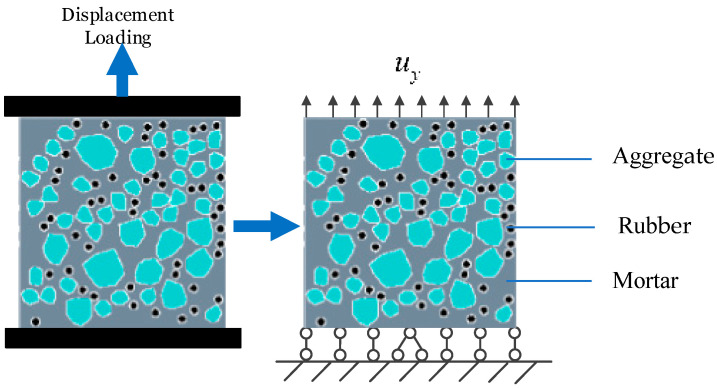
Tensile loading model.

**Figure 13:**
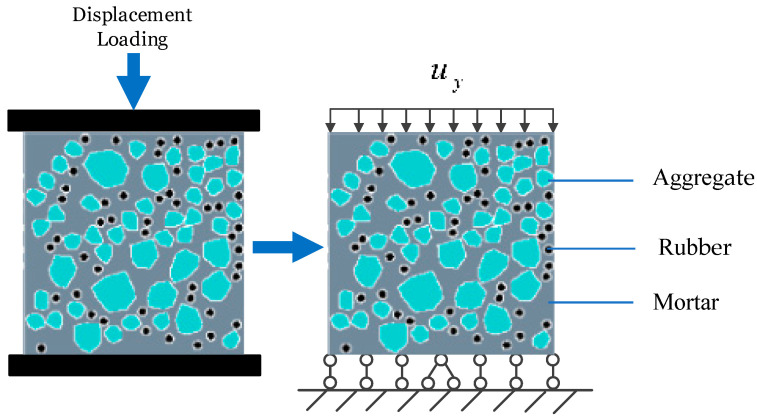
Compressive loading model.

The loading models for both the uniaxial tension and compression of RRC are established, as illustrated in Figure 12 and Figure 13, respectively. In both cases, all points at the bottom are restricted in vertical displacement to prevent rigid body movement, and the horizontal displacement of the middle node at the bottom is restricted. For the tension model, a uniform load is applied at the top, with equal displacement increment loading and a loading rate of 0.001 mm/load step. In the compression model, a uniformly distributed load is applied at the top, employing an equal displacement increment loading method but with a loading step of 0.01 mm/load step.

## 5. Failure Mechanism Results of Recycled Rubber Concrete

### 5.1. Uniaxial Tension Results

**Figure 14:**
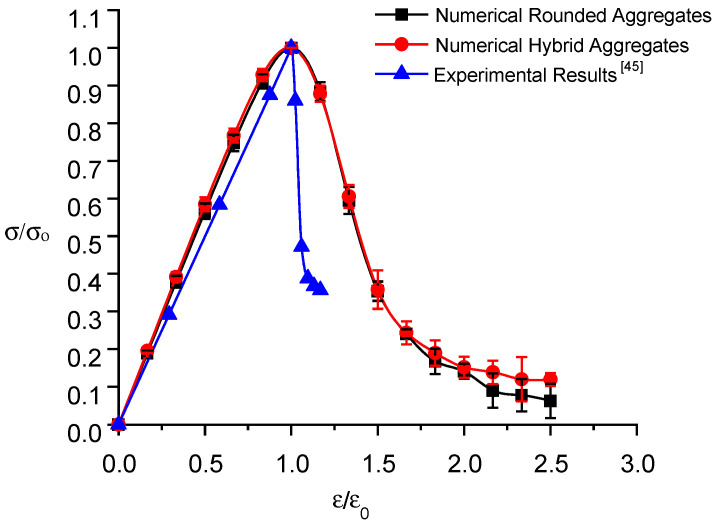
Normalized stress–strain curve of recycled rubber concrete under uniaxial tension.

Using the base force element method for the complementary energy principle, numerical simulations of uniaxial tensile tests are performed on the three specimens randomly placed in each model. The ratio of stress to peak stress, $\sigma /\sigma_{o}$, is used as the vertical coordinate, and the ratio of strain to peak strain, $\varepsilon ∕\varepsilon_{o}$, is used as the horizontal coordinate to plot the normalized stress–strain curve of the numerical simulation and experiment, as shown in Figure 14.

The calculated results of the two types of random aggregate models and the experimental data [45] are listed in Table 4.

Examination of Table 4 and Figure 14 reveals key findings related to RRC. Rounded aggregate samples exhibit peak stresses of 1.045 MPa, 1.018 MPa, and 0.951 MPa, with an average of 1.005 MPa and an error within 10%, differing by 5.5% from experimental data. Conversely, hybrid aggregate samples register peak stresses of 0.929 MPa, 1.002 MPa, and 0.949 MPa, with an average of 0.96 MPa and an error within 5%, differing by only 0.7% from the experiments. This result supports the increased accuracy of the hybrid random aggregate model and emphasizes the influence of aggregate shape on tensile strength. The uniaxial tension process consists of an elastic stage followed by damage and destruction phases, corresponding to a linear stress–strain curve and a peak followed by a decrease in stress. The stress–strain curves in the ascending segment exhibit adequate agreement with the experimental data. Different from the numerical simulation results of uniaxial tensile, the declining segment of the stress–strain curve of uniaxial compression numerical simulation decreases more slowly.

**Figure 15:**
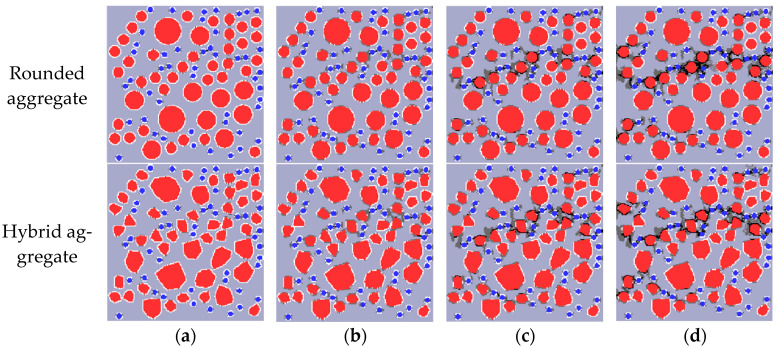
Uniaxial tensile damage diagram depicting mortar, aggregate, and rubber in grey, red, and blue, respectively: (**a**) 80% peak stress; (**b**) peak stress; (**c**) 60% peak stress (post peak); (**d**) 10% peak stress (post peak).

In order to analyze the failure mechanism and damage process of RRC under uniaxial tension, one specimen is taken from each of the two groups of models, and the Fortran program is used to draw the damage and failure diagram of uniaxial tension, as shown in Figure 15. In the figure, the mortar is light gray, the aggregate is red, the rubber is blue, the ITZ shell is white, the damaged mesh is dark gray, and the broken mesh is black.

Analysis of Figure 15 elucidates the patterns of damage, failure, and crack propagation in RRC under uniaxial tension. Initially, during loading, the specimen remains in the elastic stage until 80% peak stress, when damage around individual rubbers begins. As loading progresses, the damage extends to the aggregate ITZ, connecting with adjacent damaged areas and forming 1–2 obvious cracks. Those cracks are generally perpendicular to the loading direction, leading to a rapid decrease in stress and fast failure. This behavior conforms to the failure law of concrete under tension. Additionally, dense areas of rubber particles are the first to crack, suggesting that the strength of RRC could be improved by evenly dispersing the rubber particles during preparation. Comparatively, cracks initially appear along the longer side edge in convex polygon aggregate models and then expand outward, different from the round aggregate model.

**Figure 16:**
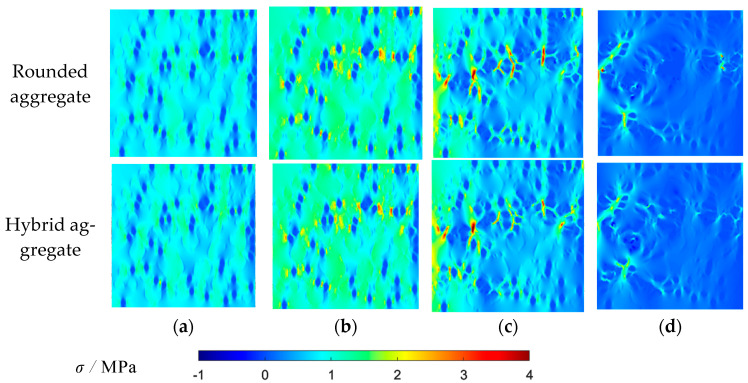
Uniaxial tensile maximum principal stress contour map: (**a**) 80% peak stress; (**b**) peak stress; (**c**) 60% peak stress (post peak); (**d**) 10% peak stress (post peak).

**Figure 17:**
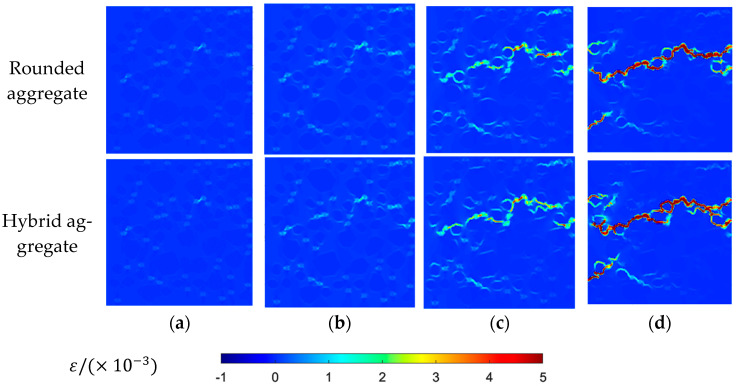
Uniaxial tensile maximum principal strain contour map: (**a**) 80% peak stress; (**b**) peak stress; (**c**) 60% peak stress (post peak); (**d**) 10% peak stress (post peak).

MATLAB-R2022A-v9.12 is used to draw the maximum principal stress contour maps and maximum principal strain contour maps of two aggregate models at different loading stages, as shown in Figure 16 and Figure 17.

Figure 16 and Figure 17 present the distributions of maximum principal stress and strain under different stress loads. Initially, the higher elastic modulus of mortar and coarse aggregate compared to rubber leads to large stress and strain around rubber particles, resulting in uneven distribution. Upon reaching peak stress, concentration phenomena occur, causing failure at the interfaces between rubber and mortar (ITZ meshes). As loading continues, stress and strain concentration intensify, leading to the failure and interconnection of cracks, particularly around rubber ITZ and aggregate ITZ. This progression culminates in the formation of a main crack, with stress decreasing but strain persisting. The observed stress distribution and crack development align fully with the features seen in the damage failure diagram, affirming the accuracy of the damage contour map.

### 5.2. Uniaxial Compression Results

**Figure 18:**
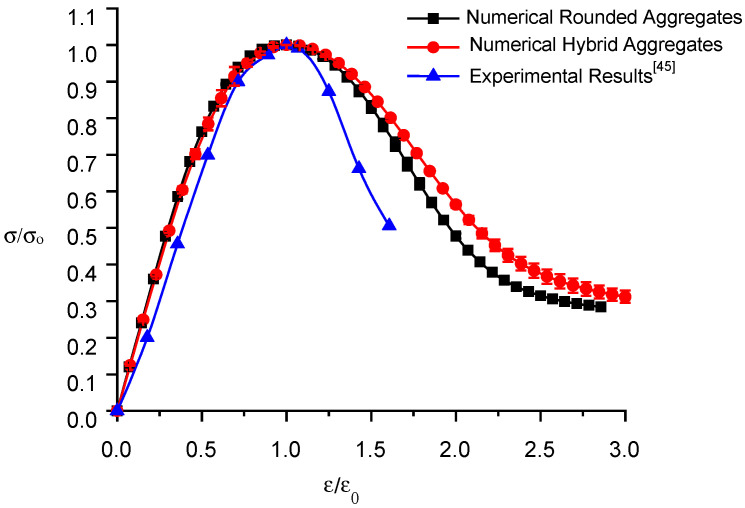
Normalized stress–strain curve of recycled rubber concrete under uniaxial compression.

Three specimens randomly placed in each model are subjected to numerical simulations of uniaxial compression tests using the BFEM founded on the complementary energy principle. A normalized stress–strain curve diagram, using the ratio of stress to peak stress ($\sigma /\sigma_{o}$) as the vertical coordinate and the ratio of strain to peak strain as the horizontal coordinate, is constructed to illustrate the numerical simulation and experimental comparison, as depicted in Figure 18.

The computational outcomes from the two random aggregate models and experimental data [45] are presented in Table 5.

Analysis of Table 5 and Figure 18 reveals the peak stresses for round aggregate specimens are 16.97 MPa, 16.44 MPa, and 16.11 MPa, with a 3.2% error margin and an average of 16.51 MPa, differing by 0.4% from the experimental data. For hybrid aggregate specimens, the peak stresses are 15.75 MPa, 15.63 MPa, and 15.98 MPa, with a 6% error margin and an average of 15.79 MPa, differing by 4% from experimental data. The round aggregate model’s numerical simulation aligns closer with experimental results, while the hybrid aggregate model’s results are slightly lower. This discrepancy may stem from a difference in the ITZ element thickness (0.5 mm in the simulation vs. less than 0.05 mm in reality), increasing the weak medium and thereby reducing compressive strength. Additionally, the shape of the aggregate affects RRC’s compressive strength, with convex aggregates lowering it.

Initially, RRC exhibits elastic behavior under uniaxial compression. As loading progresses, it enters the damage stage, with stress growth slowing and eventually decreasing after peaking, leading to a faster decline toward residual strength in the failure stage. The numerical simulation of stress–strain curves for both random aggregate models align well with experimental data in the rising segment. However, it falls slightly slower in the declining segment, potentially due to actual concrete’s higher porosity and defect rate, causing quicker brittle failure.

**Figure 19:**
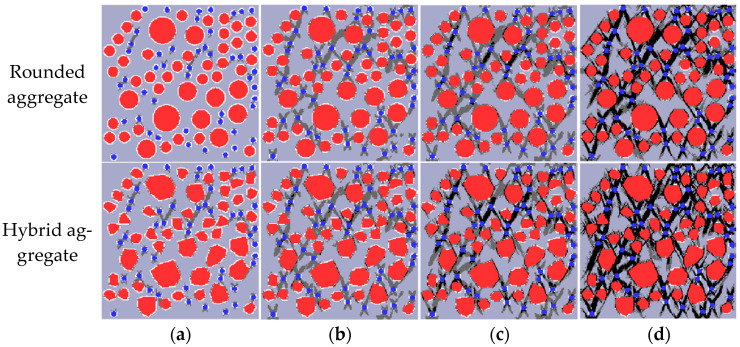
Uniaxial compressive damage diagram depicting mortar, aggregate, and rubber in grey, red, and blue, respectively: (**a**) 35% peak stress; (**b**) 80% peak stress; (**c**) peak stress; (**d**) 40% peak stress (post peak).

The failure mechanism and damage process of RRC under uniaxial compression are examined by extracting a specimen from each of the two groups of models. A uniaxial compression damage and failure diagram are subsequently created using a Fortran program, as depicted in Figure 19.

Figure 19 illustrates the damage, failure, and crack development patterns of RRC under uniaxial compression using two modeling methods. Initially, in the elastic stage, damage appears at the rubber–mortar ITZ when the load reaches 35% of the peak stress. The interface transition zone (ITZ) between coarse aggregate and mortar enters the damage state. Upon further loading to 80% of the peak stress, the mortar between adjacent rubbers fails, forming cracks. These cracks primarily develop in dense rubber particle areas, leading to longer cracks that connect at the ITZ between coarse aggregate and mortar. Near peak strength, the strength drops rapidly as cracks widen, leading to material failure and complete crushing of the specimen. Additionally, damage and cracks first appear in areas densely populated with rubber particles and small aggregates, finally distributing in the specimen in an hourglass shape at a horizontal angle of 45–60°, a feature consistent with concrete compressive failure. In comparison with round aggregates, cracks in convex polygonal aggregates initially appear around the longer edges and gradually extend and connect.

**Figure 20:**
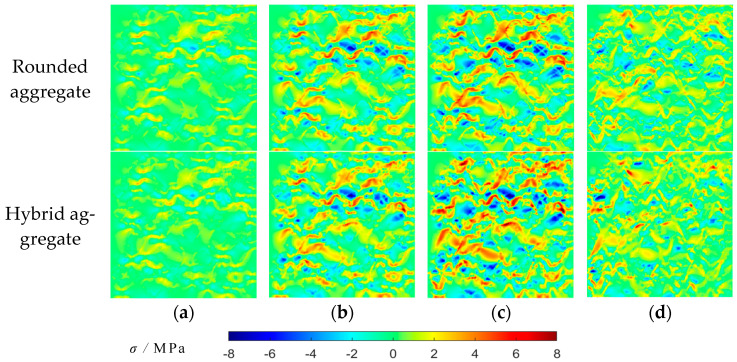
Uniaxial compressive maximum principal stress contour map: (**a**) 35% peak stress; (**b**) 80% peak stress; (**c**) peak stress; (**d**) 40% peak stress (post peak).

**Figure 21:**
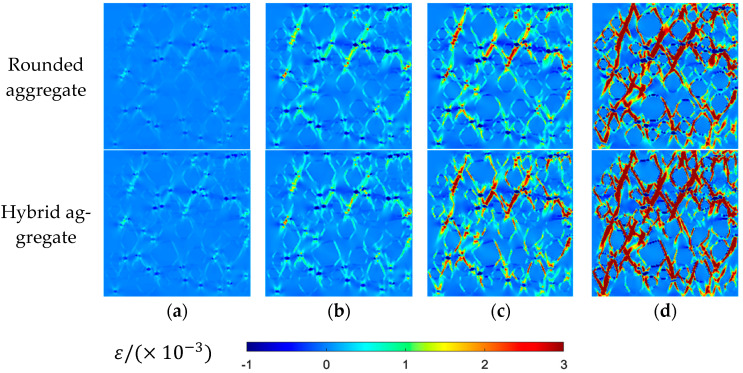
Uniaxial compressive maximum principal strain contour map: (**a**) 35% peak stress; (**b**) 80% peak stress; (**c**) peak stress; (**d**) 40% peak stress (post peak).

The MATLAB-R2022A-v9.12 software is employed to develop the maximum principal stress contour map and the maximum principal strain contour map for the two aggregate models at different loading stages. These contour maps are depicted in Figure 20 and Figure 21.

Figure 20 and Figure 21 display the distribution of maximum principal stress and strain in the model under varying stresses. The stress and strain around rubber particles are initially pronounced due to the higher elastic modulus of mortar and coarse aggregate compared to rubber. As loading advances, stress and strain concentrations lead to failure at the ITZ between rubber and mortar. Upon reaching peak stress, these phenomena escalate, causing stress and strain redistribution. This significantly increases stress and strain around the damaged meshes, initiating failure and crack expansion. The cracks interconnect and perforate, decreasing stress but increasing strain. The stress distribution contour map highlights that stress near rubber is larger; thus, rubber particles should be fully dispersed for higher compressive strength. The observed stress distribution aligns with the crack characteristics in the failure diagram, confirming the validity of the damage failure diagram.

The behavior of recycled rubber concrete (RRC) can exhibit noteworthy differences under compression and tension. Compressive loading typically results in crushing, with the initial emergence of cracks in the dense areas containing rubber particles and small aggregates, followed by their distribution throughout the specimen. On the other hand, tensile forces lead to the formation of primary cracks, which originate around individual rubbers’ interfacial transition zones (ITZs), extend to the adjacent aggregate ITZs, and develop into one or two perpendicular cracks, ultimately leading to rapid failure. The difference in ITZ thickness between the simulation and reality could play a more significant role in the compressive case, as this region could be the weakest link, thus rendering it more susceptible to crushing. However, in tension, the overall material cohesion, including the distribution of voids and aggregate sizes, may play a more substantial role in crack initiation and propagation. These findings align with the study by Guinea et al. [47], which concluded that the quality of ITZ has a strong influence on the compressive strength and modulus of elasticity compared to tensile strength. Additionally, this study provides insights into the fundamental mechanical properties of rubber concrete, where the uniaxial compressive strength is most significantly impacted by the rubber substitution ratio.

## 6. Conclusions

Recycled rubber concrete (RCA), a promising green construction material, offers solutions to environmental challenges associated with rubber waste. While its macro-mechanical properties have been extensively researched, there is a notable gap in mesoscopic analysis. In this study of RRC, an advanced hybrid random aggregate model was developed by integrating convex polygon aggregates and rounded rubber co-casting programs. This model was further supplemented by preprocessing and postprocessing tools programmed in MATLAB and Fortran. Numerical simulations, guided by the base force element method (BFEM) of the complementary energy principle, are conducted on uniaxial tensile and compressive tests of RRC using the rounded and hybrid aggregate models. Based on the analysis, the following key findings can be inferred:

- Under tension, one or two primary cracks appear perpendicular to the load direction, while compression induces bifurcated cracks angled between 45 and 60°, akin to conventional concrete’s failure patterns.
- The more irregular the shape of aggregate and the more obvious the angle, the lower the compressive strength of rubber concrete. However, when the degree of irregularity of the aggregate is lighter, that is, when it is closer to the circle, the crack will be inhibited, and the strength will be slightly increased.
- The produced stress–strain curves, mesoscopic damage process diagrams, and principal stress and strain contour maps align closely with experimental data, reaffirming the method’s credibility.
- This study shows that the base force element method based on the complementary energy principle can be used to analyze the relationship between the microstructure and the macroscopic mechanical properties of rubber concrete materials and the failure mechanism of rubber concrete materials.

Future directions for research encompass three-dimensional modeling, dynamic damage analyses, precise aggregate shape modeling, improved ITZ simulations, consideration of defects, and applications to diverse structural components in construction. It is of great theoretical significance and application value to study the mechanical properties and failure mechanism of rubber aggregate concrete by using the method of mesoscopic damage analysis.

## Figures and Tables

**Table 1:** Particle sizes range and particles count of aggregate for recycled rubber concrete.

Particle Size Range/mm	Average Particle Size/mm	Particles Count
20–15	17.5	2
15–10	12.5	9
10–5	7.5	36

**Table 2:** Material parameter.

Medium	Compressive Strength/MPa	Tensile Strength/MPa	Elastic Modulus/GPa	Poisson’s Ratio
Aggregate	100	10	55	0.16
Rubber	/	/	0.07	0.49
Mortar	24	2.95	18.97	0.22
Aggregate–Mortar ITZ	15.6	1.92	12.33	0.2
Rubber–Mortar ITZ	8.4	1.03	6.64	0.2

**Table 3:** Constitutive parameters.

Constitutive Parameters	Mortar	Aggregate	Aggregate–Mortar ITZ	Rubber–Mortar ITZ
α	0.85	0.5	0.65	0.65
β	0.2	0.2	0.2	0.2
μ	0.2	0.2	0.2	0.2
λ	0.3	0.5	0.3	0.3
$\lambda_{t}$, $\lambda_{c}$	4	5	3	3
$\eta_{t}$, $\eta_{c}$	10	10	10	10

**Table 4:** Comparison of numerical simulation results of uniaxial tension.

Specimen	Numerical Simulation Results/MPa	Simulation Results Average /MPa	Experimental Results [45]/MPa
Round Aggregate Specimen 1	1.045	1.005	0.953
Round Aggregate Specimen 2	1.018		
Round Aggregate Specimen 3	0.951		
Hybrid Aggregate Specimen 1	0.929	0.960	0.953
Hybrid Aggregate Specimen 2	1.002		
Hybrid Aggregate Specimen 3	0.949		

**Table 5:** Comparison of numerical simulation results of uniaxial compression.

Specimen	Numerical Simulation Result/MPa	Average Calculation Result/MPa	Experimental Result [45]/MPa
Round Aggregate Specimen 1	16.97	16.51	16.45
Round Aggregate Specimen 2	16.44		
Round Aggregate Specimen 2	16.11		
Hybrid Aggregate Specimen 1	15.75	15.79	16.45
Hybrid Aggregate Specimen 2	15.63		
Hybrid Aggregate Specimen 3	15.98		

## Data Availability

The data that support the findings of this study are available from the corresponding author upon reasonable request.
